# Ga-DOTA PET/CT: the first-line functional imaging modality in the management of patients with neuroendocrine tumors

**DOI:** 10.1590/0100-3984.2022.55.2e2

**Published:** 2022

**Authors:** Cláudio Tinoco Mesquita, Isabella Caterina Palazzo, Maria Fernanda Rezende

**Affiliations:** 1 Radiology Department, Universidade Federal Fluminense (UFF), Niterói, RJ, Brazil.; 2 Hospital Pró-Cardíaco, Rio de Janeiro, RJ, Brazil.; 3 Hospital Vitória e Hospital Samaritano da Barra, Rio de Janeiro, RJ, Brazil.

Neuroendocrine tumors (NETs) constitute a heterogeneous group of neoplasms that account for approximately 0.5% of all newly diagnosed malignancies. The primary sites of NETs are the gastrointestinal tract (in 65%) and the lungs (in 25%). Nearly one quarter of all NETs are metastatic at diagnosis^(1)^. Most NETs express somatostatin receptors (SSTRs) in the cell membrane, which allows the use of radiotracers that bind with high affinity to SSTRs, such radiotracers being used for diagnosis, staging, prediction of a treatment response and even as therapeutic agents^(2-4)^. The first radiotracer used in order to evaluate SSTR expression was ^123^I-labeled Tyr^3^-octreotide, which was developed in 1983^(5)^. Because of the excessive intestinal accumulation of this tracer due to high biliary excretion, there was a search for a better radiotracer, which resulted in the development of [(^111^)In-DTPA(0)]octreotide ( ^111^In-pentetreotide). In 1993, Eric Krenning’s team published data related to the use of ^111^In-pentetreotide imaging in more than 1,000 patients, demonstrating that the modality has good sensitivity and specificity^(6)^. Trials of ^111^In-pentetreotide established the pathway to developing tracers dedicated to therapy using the same mechanism of cell binding (theranostics), designated peptide receptor radionuclide therapy (PRRT), which is now considered an important option for the treatment of patients with well-differentiated NETs^(2)^.

Despite the established clinical value of using ^111^In-pentetreotide as a radiotracer, it has some shortcomings, including a high radiation burden, less than optimal spatial resolution (i.e., less ability to detect small lesions), longer image acquisition time, some accumulation in the intestine/gallbladder, and less availability^(7)^. In an article published in the previous issue of Radiologia Brasileira, Cavicchioli et al.^(8)^ compared the use of ^68^Ga-DOTA-D-Phe^1^,Tyr^3^-octreotate ( ^68^Ga-DOTATATE) positron-emission tomography/computed tomography (PET/CT) with that of ^111^In-octreotide scintigraphy in 41 patients with NETs. Images were compared in a patient-by-patient analysis to identify additional lesions and determine their impact on clinical management. In 33 patients, both exams were positive, although ^68^Ga-DOTATATE PET/CT revealed more positive sites in one third of the cases (11 patients). The results were discordant in five patients, in whom ^68^Ga-DOTATATE PET/CT was positive and ^111^In-octreotide scintigraphy was negative. In five patients, there were changes in clinical management based on the additional information obtained by ^68^Ga-DOTATATE PET/CT. The authors concluded that ^68^Ga-DOTATATE PET/CT is superior to conventional ^111^In-octreotide scintigraphy for the management of NETs because the former better discriminates the extent of disease and has greater capacity to change the treatment strategy.

How do the findings of Cavicchioli et al.^(8)^ relate to data in the literature? Their findings are in good agreement with the results of a systematic review and meta-analysis published in 2016, in which ^68^Ga-DOTATATE PET/CT was compared with ^111^In-DTPA-octreotide imaging and conventional imaging for the evaluation of pulmonary and gastroenteropancreatic NETs^(9)^. Only three of the studies evaluated in that review directly compared the two radiopharmaceuticals in the same patient, and the results were similar: for the diagnosis and reassessment of tumors with high SSTR expression, ^68^Ga-DOTATATE is more sensitive than is ^111^In-DTPA-octreotide and should be used if available. Here in Brazil, Etchebehere et al.^(10)^ compared another single-photon emission CT (SPECT) tracer with high affinity for SSTRs (^99m^Tc-HYNIC-octreotide) with ^68^Ga-DOTATATE and found that the latter seems to be more sensitive for the detection of well-differentiated NETs, especially those in bone and those that were previously unknown. Those authors suggested that SSTR-binding SPECT tracers should be used only when ^68^Ga-DOTATATE PET/CT and magnetic resonance are not available. We strongly agree with this recommendation, and we can state that ^68^Ga-DOTATATE PET/CT should be the functional imaging modality of choice in the management of patients with NETs. SPECT, preferably SPECT/CT, should be reserved for cases in which ^68^Ga-DOTATATE PET/CT is not available. The National Comprehensive Cancer Network guidelines for NETs currently recommend that SSTR imaging options should include SSTR-PET/CT or SSTR-PET/MRI, or octreotide SPECT/CT (only if SSTR-PET/CT is not available)^(11)^.

Not only is ^68^Ga-DOTATATE PET/CT more accurate than octreotide SPECT for NET localization, it also plays an important role in the selection of NET patients for treatment. In 2017, a landmark clinical trial demonstrated the benefit of the use of PRRT in patients with metastatic midgut NETs with SSTR expression^(2)^. In that study, progression-free survival was markedly longer among the patients who received ^177^Lu-DOTATATE than among those who received high-dose octreotide. Our personal experience is that PET/CT is preferred for selecting patients for PRRT because it can identify patients that have advanced disease not diagnosed by conventional imaging, thus avoiding unnecessary surgical procedures.

In addition, ^68^Ga-DOTATATE PET/CT is a marker of prognosis: increased uptake of the tracer in a tumor indicates well-differentiated neoplastic cells, which has been shown to correlate with improved overall survival^(12)^. However, ^18^F-fluorodeoxyglucose (FDG) uptake on PET has an opposite prognostic association: higher FDG uptake in tumors has consistently been associated with poorer overall survival. A recent meta-analysis showed that, in NET patients, ^18^F-FDG PET imaging prior to PRRT administration appears to be a useful tool to predict tumor response and survival outcomes, negative FDG uptake by the tumor being associated with longer progression-free and overall survival^(13)^. Based on those findings, there are classifications for clinical use that combine the FDG PET results with those of ^68^Ga-DOTATATE PET in staging subjects with NET, to provide better support for clinical management.

Novel radiotracers for SSTR imaging are under study. It has been shown that ^18^F-labeled tracers have better resolution potential than do ^68^Ga-labeled tracers because of their lower positron energy. Therefore, there is intense interest in adding a cyclotron-produced tracer to the clinical portfolio; one of the best candidates is ^18^F-AlF-NOTA-octreotide ([ ^18^F]AlF-OC), because it shows favorable kinetic and imaging characteristics^(14,15)^. Studies involving head-to-head comparisons to validate [ ^18^F]AlF-OC as a ^18^F-labeled alternative to ^68^Ga-DOTATATE in clinical applications of PET are now underway and must be completed before this tracer can be used in clinical practice. The pathway to the development of new tracers and the use of PRRT for the management of NETs is a brilliant example of the importance of the scientific effort. Patients with rare diseases such as NETs can derive great benefit from well-designed research and teamwork.
